# Assessment of Concentrated Liquid Coffee Acceptance during Storage: Sensory and Physicochemical Perspective

**DOI:** 10.3390/molecules26123545

**Published:** 2021-06-10

**Authors:** Mónica Quintero, Sebastián Velásquez, Julián Zapata, Carlos López, Luis Cisneros-Zevallos

**Affiliations:** 1 Research and Development Center—Colcafé S.A.S., Medellín 050023, Colombia; svelasquez@colcafe.com.co; 2 Department of Chemistry, University of Antioquia, Medellín 050010, Colombia; julian.zapatao@udea.edu.co (J.Z.); carlopez.udea@gmail.com (C.L.); 3 Department of Horticultural Sciences, Texas A&M University, College Station, TX 77843, USA; lcisnero@tamu.edu

**Keywords:** concentrated liquid coffee (CLC), sensory analysis, shelf-life, multivariate analysis, flavor changes

## Abstract

Concentrated liquid coffees (CLCs) refer to stored extracts stable at environmental temperature, used as ingredients in the retail market. Their low chemical stability affects the sensory profile. This study was performed in two CLCs, one without additives (BIB) and another with a mix of sodium benzoate and potassium sorbate additives (SD), stored at 25 °C for one year. Quantitative-Descriptive (QDA) and discriminant analyses permitted identifying the critical sensory attributes and their evolution over time. The concentrate without additives presented an acceptance limit of 196 days (evaluated at a 50% acceptance ratio), while the additives increased the shelf life up to 226 days (38.9% improvement). The rejection was related to a decreased aroma, increased acidity, and reduced bitterness. A bootstrapped feature selection version of Partial Least Square analysis further demonstrated that reactions of 5-caffeoylquinic acid (5CQA) and 3,5-dicaffeoylquinic acid (3,5diCQA) could cause changes in the aroma at the first degradation stage. In the following stages, changes in fructose and stearic acid contents, a key indicator of acceptance for both extracts possibly related to non-enzymatic reactions involving fructose and other compounds, might affect the bitterness and acidity. These results provided valuable information to understand flavor degradation in CLCs.

## 1. Introduction

Traditionally, coffee has been consumed as a hot beverage. The coffee market is growing in new categories, changing from hot to cold brews, ready-to-drink (RTD) products to attract millennials and centennials, and offering new consumption. Coffee acceptability depends mainly on its sophisticated flavor. Concentrated liquid coffees (CLCs) are one of these new product categories, and these concentrates are employed as ingredients in the production of canned coffee, cold brews, and other beverages based on coffee. Cold coffees are trendy in some countries like Japan, Korea, and the USA [1]. However, it is well-known that coffee products are complex matrices; especially, brews and liquid presentations are supremely unstable with respect to the changes in their sensory profile during storage, even if frozen [2].

CLCs, due to their water content, require of a pasteurized or a sterilized process to prevent microbial spoilage [3,4,5]. Furthermore, common additives such as antifungals, pH controllers, and special packages have been implemented to maintain sensory characteristics with minimal changes in the profile during the long-term storage time [6,7]. The shelf life claimed by commercial products varies between 3 and 6 months, depending on the addition or not of additives and the solid soluble contents [1]. However, their sensory profiles deviate significantly since the first month of shelf life compared to a freshly brewed roasted coffee beverage.

Research on coffee shelf life has been addressed mainly to understand the deterioration reactions in roasted and brew coffees [8,9,10,11,12]. Compared to CLCs, these beverages have total solids below 3% and are usually brewed in manual machines. Contrarily, CLCs are industrially produced and come in high total dissolved solids presentation in a range between 15% and 45%. These differences impact the sensory quality of the coffee cup and its shelf life. CLCs are classified as nonperishable food and require a deeper understanding of critical deterioration phenomena during their time storage.

Changes in flavor during storage of CLCs are an essential consideration for the development of RTD beverages and their use in specific distribution channels as vending machines. Despite its commercial use, CLCs stability, deteriorative causes, and variables that affect their sensory quality are not well known, and limited information about their chemical and physical change events that take place during storage is available [2]. Coffee deterioration is mainly related to flavor and aroma changes, presenting a vast difference in the shelf life for each product, which are classified as long-term perishables, specifically for CLCs [13].

Investigations on quality depletion in coffee beverages have shown that this event is mainly characterized by an increment of perceived sourness along with enhanced acidity seen as an increase in titratable acidity and a pH decrease. The deterioration depends on the type of processing, package, and final constituents in the matrix, like chlorogenic acids, mono-esters, carbohydrates, and volatile compounds [4,7,14,15]. Deteriorative changes in coffee brews have been reported to be related to microbial spoilage [3], oxidative reactions, volatile loss, ester hydrolysis, non-enzymatic browning [2], interactions between volatile and non-volatile coffee constituents [6,16,17]; among other mechanisms which have not been fully described. Nevertheless, the change in sourness has been identified as a critical sensory attribute in the definition of shelf life for coffee brews for a storage time below 90 days [4,15]. However, this quality stability marker has not yet been validated.

Undesired changes in the acidity and sourness have been shown to be correlated with the reduction in the 5-caffeoylquinic acid (5CQA), increases of caffeic and ferulic acids in coffee brews stored for 120 days [4,7]. Other studies suggest that these changes are the product of non-enzymatic browning (NEB) given by complex reactions between carbohydrates and amino acids, which might involve lactones hydrolysis [4,8]. Several authors widely studied volatile changes in coffee beverages, and they showed that chlorogenic acids and melanoidins are involved in aroma loss [6,16,18]. These reactions occur due to oxygen interacting with chlorogenic acids and develop adducts such as hydroxyhydroquinones that simultaneously react with thiols, especially with 2-furfurylthiol, which is the main responsible for the coffee aroma note in the beverage [17,18]. Melanoidins are active in the entrapment of thiols compounds; thus, the deteriorative changes in the aroma in the coffee brews [6,19].

One of the most conventional approaches for stability is by dosing additives; it has been widely used for the production of fruit drinks, juices, and beverages [7,20,21,22]. These additives might inhibit the reaction pathways, depending on the type of molecules involved in the deteriorative process. Some additives act as antioxidants by their capacity to scavenge free radicals and oxygen [7,22]. Another mechanism consists of the use of metal chelators, and such action inhibits hydrogen production while avoiding consecutive radical reactions in the food matrix [7].

This paper presents an exploratory study following the evolution of the sensory attributes and specific chemical compounds in two CLCs that were ultra-pasteurized (UHT) and stored for one year at 25 °C, as CLC with additives (SD) and without additives (BIB). Multivariate statistics later analyzed these different parameters to identify possible routes associated with the sensory deterioration of the stored products.

## 2. Results and Discussion

### 2.1. Survival Function

**Figure 1:**
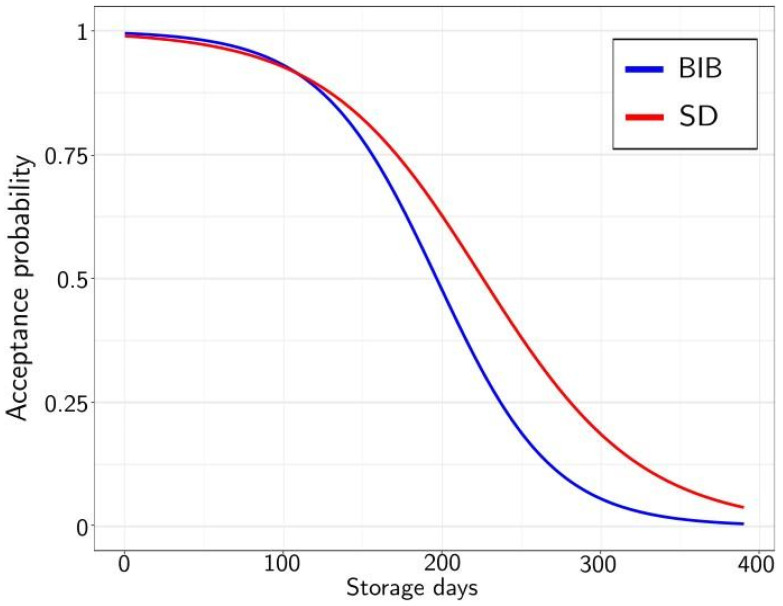
Survival analysis results of the BIB (blue) and SD (red) of acceptance probability against the storage time in days for the logarithmic model.

Figure 1 presents the survival functions for BIB and SD. The concentrate is considered rejected once the survival coefficient is below 0.5. The concentrate without additives presented an acceptance limit of 196 days (evaluated at a 50% acceptance ratio), while the additives increased the shelf life up to 226 days (38.9% improvement). This implies that additives increased the shelf life by about 30 days.

### 2.2. Sensory Attributes and Acceptability

Before starting the shelf life study, both concentrates with and without additives were compared for sensory attributes. Table 1 contains the analysis of variance (ANOVA) results for the initial QDA analysis (i.e., BIB and SD). Attributes that significantly varied with storage were aroma, acidity, and bitterness.

**Figure 2:**
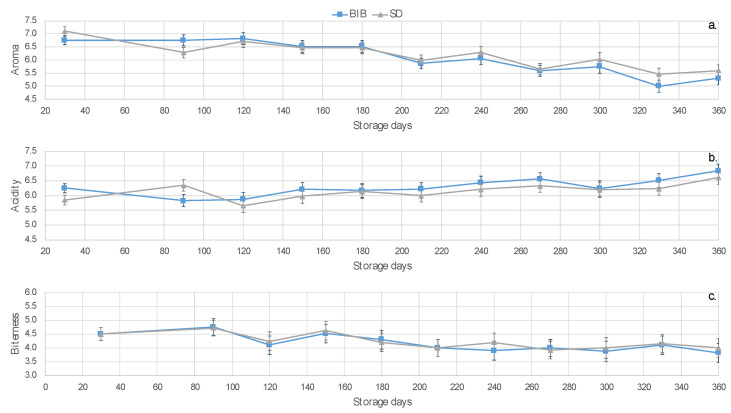
Intensities reported by panel judges (*n* = 10). Mean (solid line) and standard error (error bars) showing the changes for aroma (**a**), acidity (**b**), and bitterness (**c**) as a function of the storage time in days. Line color depicts CLC category BIB (blue) and SD (grey).

The mean and standard error values for these attributes are presented in Figure 2. As storage time increased, an evident reduction in the aroma was perceived. BIB aroma shows a steady trend for the first 120 days in contrast to SD; after this time, an accelerated decline for both BIB and SD was observed. Acidity depicted a tendency to increase through time, with some differences between BIB and SD for the first 110 days. BIB and SD bitterness presents variations with a tendency to decline. Consequently, aroma and acidity were considered potential acceptability attributes that can define quality changes during storage.

Despite these tendencies, no significant differences were found on the monthly analysis for the attributes aroma, acidity, and bitterness by t-student and Dunn tests, between the CLCs with and without additives month 2 to 12. In contrast, in the first month, the aroma was significantly different by t-student test (*p* = 0.066) and Dunn Test (0.054) at 90% confidence.

### 2.3. Acceptance Modeling

Mean and standard error values for the variable importance projection (VIP), selectivity ratio, and normalized coefficient for the variables for each CLC are included in Table 2. Along with the specified values, the number of models in which the variable was included is also presented.

Sensory attributes aroma and acidity were both used in the BIB and SD models, which agrees with the results presented in Section 2.2. Coefficients for all attributes agree with previous results. The chemical attributes that were most significant for both BIB and SD were color chroma and a*, which represent the change from green to red. For both concentrates, an increase in red is perceived; it was more pronounced in SD; hence, color can be correlated with changes in chemical composition due to shelf life. It is well known that color has been used to correlate sensory characteristics with the roasting level in coffee [11,23]. Torma et al. [24] reported that differences in coffee color are correlated with the kind of melanoidins present in the beverage, and these are products from the Maillard reaction. They found that burnt, sour, and bitter notes are strongly interlinked with these differences. The increase in fructose contents might be associated with changes in the products of Maillard reaction at acidity conditions (≤ pH 5) by the hydrolysis of low molecular weight melanoidins. Reducing sugars present reversible reactions with amines to produce glucosamine, which undergoes an Amadori rearrangement to give D-glucose a derivative of 1-amino-1-deoxy-D-fructose. The formation of Strecker aldehydes might accelerate degradation pathways in CLCs during the UHT processing and advanced glycation end products (AGEs), which are modifications of lysine residues [6,25]. There is not enough evidence in the literature to explain these mechanisms, nor the relationship between color degradation with carbohydrate contents during storage in coffee beverages. Further studies may be conducted to understand and find strategies to maximize shelf life stability. However, these results suggest that color might be used as an indicator of shelf life.

The chlorogenic content expressed by 5CQA and 3,5diCQA were relevant to explain CLC acceptance in BIB through time. The concentration of 5CQA increased during storage in CLC coffee brews stored for 90 and 120 days as reported by Sopelana et al. [4]. They suggested that these changes result from the hydrolysis of chlorogenic acid lactones or to the release of CQAs from non-covalently linked polymeric skeletons, such as melanoidins. Furthermore, this work suggests that changes in color and increases in fructose might be more related to the second assumption: Nevertheless, 3,5diCQA increase contents has not been previously reported. Interestingly, 4CQA showed an opposite effect between the two concentrates, which might have been caused by the additive employed for stability.

**Figure 3:**
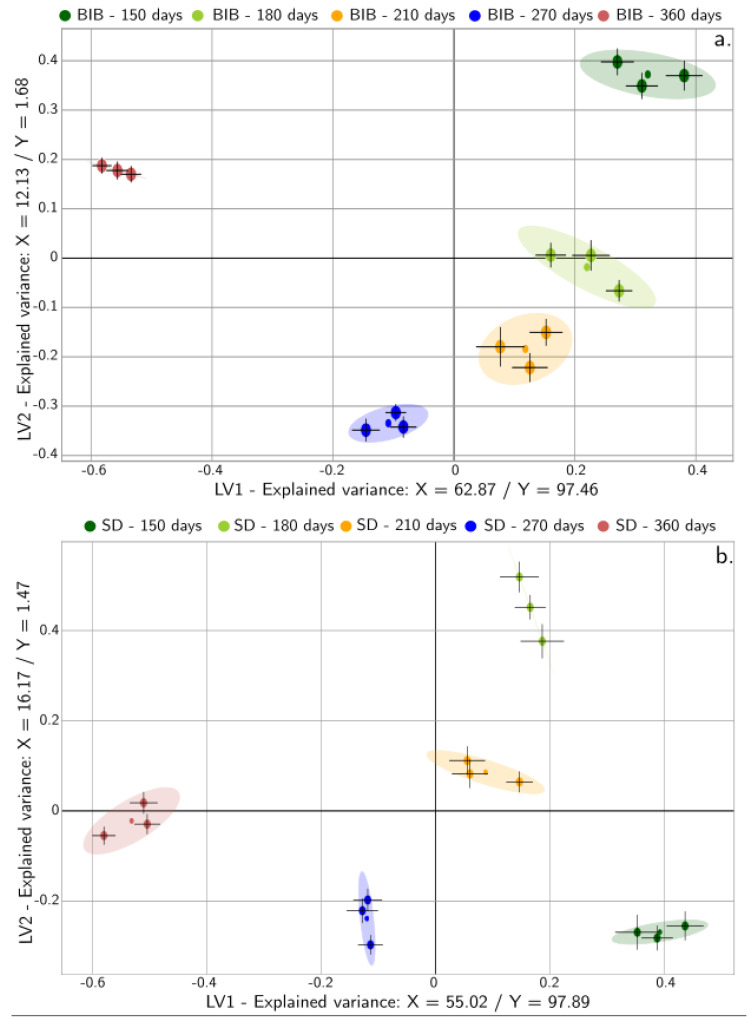
Score plot for the first two latent variables (LV1/LV2) for the acceptability model of the two CLCs: (**a**) BIB and (**b**) SD for the multivariate feature—acceptability model.

**Figure 4:**
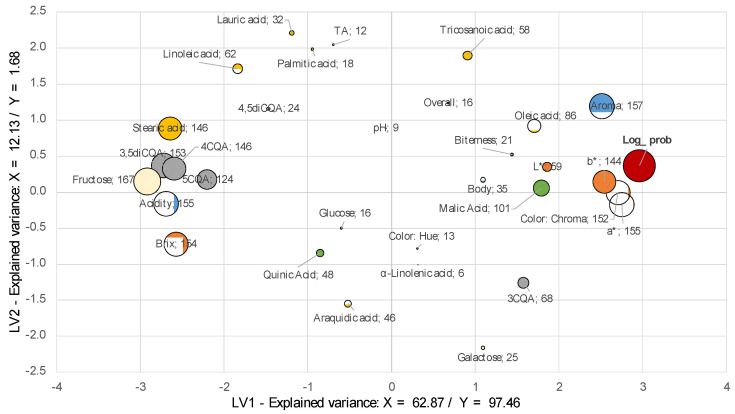
Loadings plot for BIB concentrate samples, storage during 12 months at 25 °C by boo -strapped—feature selection version of PLS analysis. Color depicts feature type: red—log-acceptability ratio, blue—sensory, gray—CQAs, yellow: organic acids and orange—physicochemical; bubble size represents variable relevance.

**Figure 5:**
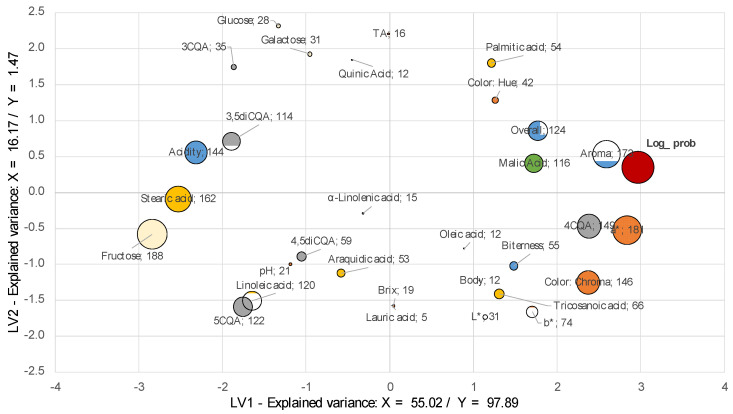
Loadings plot for SD concentrate samples, storage during 12 months at 25 °C by bootstrapped—feature selection version of PLS analysis. Color depicts feature type: red—log-acceptability ratio, blue—sensory, gray—CQAs, yellow: organic acids and orange—physicochemical; bubble size represents variable relevance.

To summarize the effects, the scores plot for the first two latent variables is presented in Figure 3. For both concentrates, the first latent variable represented a variance for the input matrix (i.e., X), which ranged between 5% and 63%. Contrarily, the first variable always represented more than 55% of the variability of the output. Hence, the positive side represents samples that are accepted by the judges, while rejection is associated with the negative side of this latent variable. Latent variable 2 represents reactions that are accelerated on the first storage days, and that balance for latter storage days. The error bars represent the standard error calculated from the coefficients and variable relevance results; while the loadings are depicted in Figure 4 and Figure 5. Table 3 includes the statistics of the fitted models. All models, for both the training and test sets, had a minimum determination coefficient of 0.98.

According to Figure 4 and Figure 5, CLCs were accepted (Log-prob) when aroma had the maximum score; once this attribute decreased the staling of the CLCs is triggered, as the increase of undesirable acidity and off-flavors are more preponderant. Critical attributes associated with deterioration of the CLCs depend on the process conditions as roasting level, extraction yield, concentration and ultrapasteurization parameters. For this study, the critical sensory attributes that define the end of the shelf life were aroma in the first stage, followed by undesirable acidity. These results reveal the importance of the aroma attribute, which will be necessary to include as a sensory marker for the flavor deterioration during the storage of CLCs.

Aroma reduction in coffee beverages has been described by the reduction of roast and coffee-like notes [6,16,26]. Hofmann et al. and Charles-Bernard et al. [6,18] demonstrated that the mechanisms to explain the chemical degradation of volatile thiols and the interactions between volatile and non-volatile components result in reduction of coffee aroma notes in the brews. Accordingly, the occurrence of such phenomena in CLCs is similar. Reactive quinones released from chlorogenic acids present in the coffee matrix react with volatile thiols to form conjugates that precipitate and can be part of melanoidins. Transition metals accelerate this reaction impacting roast and coffee notes in the aroma global [18,26].

Consequently, Figure 5 shows differences for SD probably as an effect of the additives employed, with the extension of shelf-life. Latent Variable 1 (LV1) classified the variables associated with the acceptance (Log-Prob) in the positive side. Variables as the aroma, chroma, a* and 4CQA were associated with the concentrate’s acceptance. Further studies are required to clarify the effect of the additive on the increase of 4CQA.

Although it is well known that sodium benzoate and potassium sorbate act as active agents against yeast and molds, potassium sorbate has exhibited antioxidant properties by radical scavenging free radicals in aqueous solutions. Charles-Bernard et al. [6] demonstrated that the stabilizing effect of aliphatic thiols was stronger in the absence of oxygen than in the presence of ascorbic acid. Nevertheless, their findings with benzylic thiols suggested that they might undergo parallel reactions, which can be slowed by oxygen absence and radical scavengers such as ascorbic acids.

For SD, variables are presented in two groups in the negative side, in which linoleic acid and 5CQA appear as important variables. The second group is confirmed by fructose, stearic acid, and acidity. The hypothesis for this change is the additives that might operate as antioxidants and pH regulators, minimizing the deterioration for additional 30 days.

The antioxidant effect of potassium sorbate might explain the differences in the shelf life by over 30 days between BIB and SD concentrates by avoiding the deteriorative reaction produced by the oxygen present in the package headspace. The analysis performed in the headspace of the packages showed that the bags contain 14% of oxygen and 40% of carbon dioxide contents in the headspace. Further studies should be conducted in order to understand the role of these gases in deterioration reactions.

## 3. Materials and Methods

### 3.1. Chemicals and Reagents

The methanol and acetonitrile, supra-gradient high-performance liquid chromatography (HPLC) grade, was provided by Merck (Darmstadt, Germany). Pure reference standards of 5-caffeoylquinic acid (5CQA), 4-caffeoylquinic acid (4CQA), 3-caffeoylquinic acid (3CQA), 3,4 di-caffeoylquinic acid, 3,5 di-caffeoylquinic acid, 4,5 di-caffeoylquinic acid, were purchased from Phytolab (Núremberg, Germany); malic acid and quinic acid from Chromadex (Irvine, USA); caffeic acid, fructose, and glucose were obtained from Sigma Aldrich Co. (St. Louis, MO, USA). Fatty acids methyl esters mix was purchased from Sigma Aldrich Co. (St. Louis, MO, USA) to determine the respective lauric acid, palmitic acid, stearic acid, oleic acid, linoleic acid, α-linolenic acid, arachidonic acid and tricosanoic acid contents.

### 3.2. Preparation of Concentrated Liquid Coffee (CLC)

The production of CLC was made from roasted ground coffee at color level L* = 20.00 ± 0.4 in an industrial system (Colcafé S.A.S., Medellín, Colombia) for the production of instant coffee. The process comprised a percolation battery with six extractors, and each one contained 300 Kg of coffee; then vapor at 180 °C passed through the coffee at constant flow to extract the coffee liquid. In this part of the process, the concentration rises to a range of 10–15% soluble solids. Then, the liquid passed through to an industrial evaporator, and the soluble coffee solids raised to 30–45%. Finally, CLC was subjected to ultra-high pasteurization treatment at 121 °C for 5 s to avoid microbiological spoilage and packing.

Specific process conditions for the CLCs in this study were called BIB, which refers to liquid concentrate without additives. SD liquid concentrate, which previous to the UHT process, were added sodium benzoate (E-211) and potassium sorbate (E-202) at 500 mg/Kg, each one. These additives are approved by the Codex Alimentarius and are commonly used as food preservatives with a broad spectrum action against yeast and molds [22]. The mixes of both at a maximal level have shown a synergistic action in fruit juices by antimicrobial effect [20,21,27]. Studies suggest that benzoic acid significantly protects ascorbic acid, which has exhibited antioxidant properties [20,22].

CLCs were packaged and stored in an aseptic bag with the following characteristics: 2 L of capacity, a double layer where an external film of polyethylene (PE)/polyethylene terephthalate (PET)/PE, and the internal film comprise a PE/ethylene-vinyl-alcohol (EVOH). The specifications for the transmission rate in this package system were oxygen transmission of 0.16 cc/m^2^/day at 25 °C and water vapor permeability of 11 g/m^2^/day. Before the storage, the contents of oxygen in the package’s headspace and dissolved were determined.

### 3.3. Shelf Life Design

CLCs samples were stored for 12 months. Sampling controls were kept at −38 °C and analytical samples at 25 °C. Sensory and chemical analyses were conducted according to Section 3.6, each month by duplicate. Additionally, microbiological analyses were performed every four months to guarantee food safety.

### 3.4. Coffee Beverages Preparation

Coffee beverages were prepared from CLCs with water at 90 ± 2 °C at pH 7.0 to obtain 2% soluble solids beverages. The freshly prepared coffee brews were prepared and evaluated immediately.

### 3.5. Analytical Methods

#### 3.5.1. Microbiological Analysis

Aerobic mesophilic flora and mesophilic aerobic sporulates were analyzed by a colony count technique at 30 °C in Plate Count Agar (Biolife, Milano, Italy). Enumeration of molds and yeasts was made by a colony count technique at 25 °C in oxytetracycline–glucose yeast extract agar (Oxoid, Basingstoke, UK). Enumeration of total coliforms and fecal coliforms by a colony count technique at 25 °C was considered. These analyses were performed every four months [28].

#### 3.5.2. pH and Titratable Acidity

The pH and titratable acidity analyses were performed at 25 °C by a Mettler Toledo DL 22 pH-meter automatic system (Columbus, OH, USA). Titratable acidity was determined in 100 mL of the beverage until neutrality was reached (pH 7.00) with 0.1 N NaOH.

#### 3.5.3. Total Dissolved Solid Content (TDS)

TDS was determined by a relationship between the measured Brix value, which represents the concentration of sucrose in the sample. The measurements were carried out in a Mettler Toledo R50 refractometer (Columbus, OH, USA).

#### 3.5.4. Color

The beverage color was measured with a Hunterlab D25 LT Colorimeter (Reston, VA, USA). Before each measurement, the instrument was calibrated using white and a green tiles. Color results are expressed as rectangular coordinates L*, a* and b* in CIELab parameters, in which L* indicates the degree of luminosity of whiteness or blackness (from 0 to 100). In the chromatic portion of the color, a* represents color changes between red (+a*) to green (−a*) ratio, and b* indicates the blue (−b*) to yellow (+b*) ratio. The Hue describes overall intensity while the chroma (saturation) may be defined as the strength or dominance of the hue. Equations (1) and (2) depict the calculation of both parameters.

(1) $$Hue=\pm arctan\;\left(\frac{b^{*}}{a^{*}}\right)$$

(2) $$Chroma=+\sqrt{{\left(a^{*}\right)}^{2}+{\left(b^{*}\right)}^{2}}$$

#### 3.5.5. Chlorogenic Acids Content

The content of CGAs following DIN 10767/1994: Analysis of coffee and coffee products-determination of chlorogenic acid content in roasted coffee and extracted coffee by High-Performance Liquid Chromatography (HPLC) and adapted to the coffee extracts [29]. An Agilent HPLC 1260 system (Agilent Technologies, Palo Alto, CA, USA) comprised a diode array detector (DAD) at 325 nm wavelength, a quaternary pump, and an automated sample injector. A reversed-phase Zorbax Eclipse XDB, C18, 4.6 × 150 mm, and 3.5 μm column was used for chromatographic separation. The mobile phase was acetonitrile/water (5:85, *v*/*v*) in gradient conditions at a constant flow of 1.0 mL/min and temperature at 35 °C.

Sample preparation consisted of 1 mL of CLC of CGAs, diluted with 9 mL of extraction solution (50/50 *v*/*v*: methanol/water-formic acid at 1%). Then, centrifugation for 10 min at 45,000 revolutions per minute (Hettich, Tuttlingen, Germany). The supernatant was filtered through a 0.45 μm membrane filter (Acrodisc Syringe Filter, Gelman Sciences, Ann Arbor, MI, USA) and injected into the HPLC system. The quantification made by the standard external method is expressed as the content of individual CGA per gram of extract.

This method permits the separation of seven compounds: 5-caffeoylquinic acid (5CQA), 3-caffeoylquinic acid (3CQA), 4-caffeoylquinic acid (4CQA), caffeic acid (CA), 3,4-dicaffeoylquinic acid (3,4diCQA); 4,5-dicaffeoylquinic acid (4,5diCQA), and 3,5-dicaffeoylquinic acid (3,5diCQA). External standards were employed for quantification. Each sample was measured in triplicate.

#### 3.5.6. Carbohydrate Analysis

Sugar profile analysis of the CLCs was achieved by High-Performance Liquid chromatography coupled to an Anion-Exchange with Pulsed Amperometric Detection (HPAEC-PAD), using the method described by standard ISO 11292/1995 [30]. Total carbohydrates were extracted from CLCs, 0,3 mg of concentrated was diluted with 50 mL of hydrochloric acid 1 mol/L (Sigma Aldrich Co., St. Louis, MO, USA), this solution was swirled and boiling for 150 min. Then, this dilution was filtered by 0.45 µm Nylon membrane and injected 20 µL in the liquid chromatography. Carbohydrates such as sucrose, glucose, fructose and galactose were identified and quantified by comparison with their retention times and areas of the corresponding peaks obtained for the external standard solution.

#### 3.5.7. Fatty Acid Analysis

The analyses were carried in an Agilent 7890B gas chromatograph (Santa Clara, CA, USA) coupled to a flame ionization detector (FID) (Santa Clara, CA, USA) and a 7963 auto sampler (Santa Clara, CA, USA). Injector temperature was maintained at 260 °C. The injection mode used was split 50:1 at 280 °C with a pressure pulse of 30.9 psi. Hydrogen was used (analytical grade 5.0) as the carrier gas at a constant flow of 1 mL/min during the entire analysis. The chromatographic column used was a 30.0 m TRBWAXOMEGA with 0.25 mm inner diameter and 0.25 µm film thicknesses (Barcelona, Spain). A temperature gradient carried out the separation, the initial temperature of the chromatographic oven was 225 °C which was held for 5 min; then, the temperature was increased at a rate of 15 °C/min for 2 min and posteriorly up to 240 °C at 10 °C/min for 12 min. Sample preparation 500 mg of CLCs were derivatized with boron trifluoride in methanol to convert the fatty acids in their respective methyl esters following the AOAC official method 969.33 [28].

Gas chromatographic peaks of FAME (Fatty acids methyl esters) were identified by comparing the retention time of certified standards of their ester form by derivatization (C:12 lauric acid, C:16 palmitic acid, C16:1 palmitoleic acid, C18:0 stearic acid, C18:1n9c oleic acid, C18:2n6c linoleic acid, C20:0 arachidonic acid, C18:3n3 α-linolenic acid, C23:0 tricosanoic acid). Peak areas were used for the quantification of fatty acids.

#### 3.5.8. Organic Acid Analysis

HPLC-DAD was used to determine malic, quinic, and acetic acids as described above with detection at 226 nm. The mobile phase consisted of 100 mM potassium phosphate buffer at pH 2.5 with a flow of 0.4 mL/min at 40 °C. The separation was achieved with an Agilent Poroshell 120 SB-Aq 3 × 100-mm, 2.7-µm column (Santa Clara, CA, USA). The injection volume was 3 µL. Identification was performed by comparing the corresponding retention times with their respective analytical standards and the quantitation performed by calculating areas for each peak.

#### 3.5.9. Oxygen and Carbon Dioxide Contents

The measurements of oxygen (O_2_) and carbon dioxide (CO2) contents in the headspace were determined using Moccon 325 analyzer (Minneapolis, MN, USA). The lower limit of detection with this instrument was c.a. 0.1% and a resolution of 0.01%.

### 3.6. Sensory Analysis

Ten judges were selected among the Colcafé S.A.S. specialized panel, with an age range was between 30 and 55 years. They were trained in discriminative and descriptive testing for at least 100 h previous to the real analysis using the Quantitative Descriptive Analysis^®^ (QDA) technique [31]. During the tasting sessions, 20 mL of coffee was served in 50 mL of odorless plastic cups at 70 °C. The samples were codified with randomized 3-digit numbers. Water was used for palate cleaning clean between samples. The QDA analysis consisted of evaluating the following attributes: aroma, acidity, bitterness, body and global. The intensity of each descriptor was scored on a scale from 0 to 10. The aim of this analysis was to evaluate the relationships between the evolution of these descriptors in the CLCs stored at 25 °C.

In the discriminative testing, the judges were asked to reject or accept the coffee beverage based on the sensory profile for the control sample (stored at −38 °C). In order to perform the data analysis, the beverage acceptance was coded with the number one and rejection with zero.

The evaluations were carried out in individual cubicles at 25 ± 2 °C and relative humidity in a range of 50–65%. All analyses were performed following ISO 6658:2005 standard [32]. Fizz sensory software 2.47 V was used for data acquisition.

### 3.7. Statistical Analysis

#### 3.7.1. Acceptability Modeling: Survival Function

Acceptability was considered as a classification problem; hence, the survival function was modeled by a generalized linear model considering a binomial distribution. The judges of the cupping panel were considered as the random effect, in both the intercept and the slope, and the storage time in days as the fixed effect. This model was studied for each of the concentrates: BIB and SD.

#### 3.7.2. Sensory QDA: Storage

The logarithmic version of the survival probability was calculated according to the model presented in Section 2.1. This parameter was named Log-prob, (i.e., $Log-prob=\frac{p}{1-p}\;$)), with p as the probability estimated from the survival function at each storage time. A linear mixed-effect model with this log-prob as the output was executed to evaluate the correlation of the acceptability with the sensory attributes. Consequently, the QDA sensory attributes were treated as the independent variables, and the panel as the random effect to correct the intercept for the judge effect. The analysis-of-variance (ANOVA) of the model allowed for the identification of significant attributes, followed by least squares mean analysis for mean and standard error estimation at each storage time for each of the concentrates.

#### 3.7.3. Multivariate Analysis: Physicochemical, Non-Volatile, and Sensory Representation

For the purpose of condensing all considered effects, all variables in a single model for both numerical and graphical representation were included in a bootstrapped—feature selection version of PLS [33,34]. This bootstrapping consisted of random separation of multiple training datasets, stratified by the category defined by the concentrate type and storage time variables and log-prob as the model output. Accordingly, two-thirds of the set were used for training and the whole dataset for validation purposes. As the most extensive set consisted of 15 observations, this process was necessary to accurately estimate the effect of each variable in the new latent variable representation. This approach was considered in three steps. The first stage consisted of the random generation of 100 training sets. Then, for each set, regular 1-PLS was applied. All variables were mean-centered and scaled prior to analysis, and cross-validation with 1 sample considered to select the optimal latent variable number. Although this cross-validation strategy may be inaccurate, the bootstrapping technique sought to alleviate this inconsistency by having a wide data matrix for model validation. After this first PLS run, backward feature selection was performed. The variable-importance projection (VIP) coefficient was calculated and chosen as inclusion criteria. The resulting vector was sorted in an ascending order, to remove low importance variables first. If the root mean squared error (RMSE) was maintained or decreased, the variable was removed from the model. This was iteratively performed until all variables with VIP values below one were tested. Then, the model was used for prediction with the complete dataset. This was performed to compute the projection of all observations (scores) in the new coordinate system. The scores plot includes the covariance ellipse to separate across the categories defined in the stratification. A variable relevance coefficient was computed by multiplying the mean VIP coefficient by the number of times the variable was selected by PLS. This coefficient was used to represent the size of the loadings in the loadings plot.

## 4. Conclusions

CLCs are promissory products to meet the fast dynamics involved in the actual coffee market. Nevertheless, their stability is challenged, especially for the complex chemical composition and lack of available information. Deterioration reactions for CLCs stored at 25 °C first trigger an aroma reduction, followed by an increase of undesirable acidity, which is essential to understand the development of these sensory attributes and their relationship with the chemical composition of the product. This study suggests that sensory profile changes occur by two pathways. The first one involves chemical mechanisms that result in changes of chlorogenic acids 5CQA, 4CQA, and 3,5diCQA. The results agreed with studies that showed that chlorogenic acids react with thiols responsible for the strength of the roasted notes present in the beverage [16,17,18,25,26,35]. Oxygen and transition metals accelerate these reactions as a consequence, and the aroma is impacted [15,25,26]. It is vital to understand the role of oxygen and its effect on flavor deterioration and the relationship with the changes in chlorogenic acids, fructose, and fatty acids in further studies.

The second mechanism might be associated with melanoidin hydrolysis, which can release fructose that affects the color and undesirable acidity. The addition of common additives as sodium benzoate and potassium sorbate extended the acceptance by 30 additional days. Nevertheless, the effect of the additive on 4CQA has to be further studied. This work suggests possible degradation pathways in CLCs; however, more research must be conducted to support the suggested hypotheses because of the exploratory nature of the present study.

## Figures and Tables

**Table 1:** Comparison for sensory attributes evaluated in CLCs without additive (BIB) and with additive (SD at the initial time by ANOVA.

BIB & SD			
Attributes	Mean Square	F-Value	Pr (>F)
Aroma	35.41	57.9	0.000
Acidity	3.20	5.2	0.024
Bitterness	3.95	6.5	0.012
Body	0.63	1.0	0.312
Overall	0.34	0.6	0.459

**Table 2:** Selection of variables included in the bootstrapped—feature selection version of the Partial Least Square (PLS) model. The column Models presents the number of times each variable was considered by the model, and the mean and standard error (SE) for VIP, selective ratio scores (SR), and the computed coefficient for BIB and SD.

Variable	BIB							SD						
	Models	VIP		SR		Coefficient		Models	VIP		SR		Coefficient	
	# Use	Mean	SE	Mean	SE	Mean	SE	# Use	Mean	SE	Mean	SE	Mean	SE
Aroma	100	1.57	0.05	3.92	0.81	0.14	0.04	100	1.73	0.05	3.74	1.09	0.11	0.02
Acidity	100	1.55	0.05	4.23	1.23	−0.08	0.03	100	1.44	0.14	1.32	0.80	−0.04	0.06
Biterness	25	0.85	0.25	0.44	0.39	0.04	0.02	60	0.92	0.23	0.31	0.18	0.03	0.05
Body	54	0.64	0.22	0.20	0.19	0.04	0.03	19	0.62	0.28	0.15	0.14	0.02	0.02
Overall	31	0.51	0.16	0.09	0.07	0.04	0.04	100	1.24	0.08	0.68	0.16	0.07	0.03
TA	27	0.44	0.10	0.02	0.01	0.02	0.02	51	0.32	0.06	0.01	0.01	0.04	0.01
Brix	100	1.54	0.04	3.52	0.34	−0.11	0.03	71	0.27	0.08	0.00	0.00	−0.07	0.04
pH	33	0.26	0.18	0.03	0.05	−0.03	0.05	26	0.82	0.10	0.22	0.07	−0.05	0.02
Color: Chroma	100	1.52	0.06	4.08	1.67	0.06	0.03	100	1.46	0.07	1.27	0.23	0.09	0.01
Color: a*	100	1.55	0.07	5.41	3.94	0.07	0.03	100	1.81	0.04	5.76	0.74	0.13	0.02
Color: b*	100	1.44	0.06	2.47	0.76	0.05	0.04	73	1.01	0.15	0.38	0.15	0.06	0.02
Color: L*	56	1.06	0.07	0.63	0.18	0.02	0.06	48	0.65	0.20	0.13	0.09	0.04	0.02
Color: Hue	44	0.30	0.14	0.02	0.03	0.03	0.05	48	0.88	0.13	0.27	0.10	0.05	0.01
3CQA	81	0.83	0.11	0.30	0.11	0.06	0.03	34	1.03	0.05	0.37	0.05	−0.03	0.02
3,5diCQA	100	1.53	0.04	3.67	0.68	−0.07	0.02	98	1.17	0.06	0.55	0.10	−0.07	0.02
4CQA	100	1.46	0.06	2.68	0.98	−0.07	0.02	100	1.49	0.07	1.39	0.26	0.09	0.02
4,5diCQA	31	0.76	0.15	0.24	0.11	0.01	0.04	78	0.76	0.09	0.18	0.05	−0.07	0.02
5CQA	100	1.24	0.07	1.10	0.31	−0.05	0.02	98	1.24	0.13	0.72	0.25	−0.07	0.02
Lauric acid	55	0.58	0.05	0.10	0.01	0.00	0.02	38	0.14	0.05	0.00	0.00	0.00	0.01
Palmitic acid	40	0.46	0.05	0.06	0.01	0.00	0.01	59	0.92	0.03	0.27	0.01	0.07	0.01
Stearic acid	100	1.46	0.04	2.36	0.11	−0.09	0.02	100	1.62	0.03	2.17	0.12	−0.10	0.01
Oleic acid	82	1.05	0.04	0.57	0.05	0.08	0.02	23	0.52	0.02	0.07	0.00	0.02	0.02
Linoleic acid	65	0.95	0.02	0.41	0.02	−0.04	0.01	100	1.20	0.03	0.58	0.01	−0.10	0.02
α-Linolenic acid	29	0.21	0.11	0.00	0.00	−0.02	0.03	64	0.23	0.03	0.01	0.00	0.01	0.02
Araquidic acid	95	0.49	0.04	0.06	0.00	−0.06	0.02	96	0.55	0.04	0.07	0.01	−0.08	0.02
Tricosanoic acid	82	0.71	0.04	0.17	0.01	0.08	0.02	83	0.80	0.03	0.19	0.01	0.07	0.02
Fructose	100	1.67	0.04	12.03	1.02	−0.12	0.03	100	1.88	0.04	11.78	0.74	−0.14	0.01
Galactose	46	0.54	0.04	0.08	0.01	0.01	0.02	58	0.53	0.03	0.07	0.00	−0.04	0.01
Glucose	40	0.39	0.03	0.05	0.01	−0.03	0.02	40	0.71	0.02	0.13	0.00	−0.03	0.01
Malic acid	96	1.05	0.03	0.56	0.03	0.08	0.02	100	1.16	0.03	0.53	0.01	0.09	0.01
Quinic acid	82	0.58	0.03	0.11	0.01	−0.06	0.02	47	0.26	0.08	0.01	0.00	0.01	0.01

**Table 3:** Performance statistics for the Partial Least Square (PLS) based acceptability model for both training and test datasets—R^2^ and Root Mean Squared Error (RMSE).

	BIB		SD	
	Mean	SE	Mean	SE
RMSE Train	0.106	0.043	0.067	0.024
R^2^ Train	0.996	0.005	0.999	0.001
RMSE Test	0.102	0.039	0.064	0.012
R^2^ Test	0.987	0.010	0.992	0.003

## Data Availability

The data presented in this study are available on request from the corresponding author (pending privacy and ethical considerations).

## Sample Availability

Samples of the compounds are available from the authors.
